# Effects of Sjogren’s syndrome and high sugar diet on oral microbiome in patients with rampant caries: a clinical study

**DOI:** 10.1186/s12903-024-04150-8

**Published:** 2024-03-21

**Authors:** Yifei Tang, Hua Nie, Yu Zhang, Yuan Wei, Yequan Huang, Yuan Zhuang, Weidong Yang, Yanan Zhu

**Affiliations:** Department of Endodontic, Nanjing Stomatological Hospital, Affiliated Hospital of Medical School, Research Institute of Stomatology, Nanjing University, Nanjing, China

**Keywords:** Rampant caries, Saliva, Dental plaque, Microbiota, Microbiological test

## Abstract

**Objective:**

The purpose of this study was to assess the composition of the oral microbial flora of adults with rampant caries in China to provide guidance for treatment.

**Patients and methods:**

Sixty human salivary and supragingival plaque samples were collected. They were characterized into four groups: patients with rampant caries with Sjogren’s syndrome (RC-SS) or high-sugar diet (RC-HD), common dental caries (DC), and healthy individuals (HP). The 16S rRNA V3-V4 region of the bacterial DNA was detected by Illumina sequencing. PCoA based on OTU with Bray–Curtis algorithm, the abundance of each level, LEfSe analysis, network analysis, and PICRUSt analysis were carried out between the four groups and two sample types. Clinical and demographic data were compared using analysis of variance (ANOVA) or the nonparametric Kruskal–Wallis rank-sum test, depending on the normality of the data, using GraphPad Prism 8 (P < 0.05).

**Results:**

OTU principal component analysis revealed a significant difference between healthy individuals and those with RC-SS. In the saliva of patients with rampant caries, the relative abundance of *Firmicutes* increased significantly at the phylum level. Further, *Streptocpccus, Veillonella, Prevotella,* and *Dialister* increased*,* while *Neisseria and Haemophilus* decreased at the genus level. *Veillonella* increased in the plaque samples of patients with rampant caries.

**Conclusion:**

Both salivary and dental plaque composition were significantly different between healthy individuals and patients with rampant caries. This study provides a microbiological basis for exploring the etiology of rampant caries.

**Clinical relevance:**

This study provides basic information on the flora of the oral cavity in adults with rampant caries in China. These findings could serve as a reference for the treatment of this disease.

**Supplementary Information:**

The online version contains supplementary material available at 10.1186/s12903-024-04150-8.

## Introduction

Dental caries, also known as tooth decay, is one of the most frequent chronic infectious diseases, affecting 80–90% of the world's population [1]. Rampant caries, the most serious type of caries, are characterized by multiple active carious lesions occurring in the same person and are difficult to cure [2]. Carious lesions occur on smooth surfaces on the buccal and lingual areas of the teeth, where dental caries rarely develop [3]. The cause of rampant caries is unclear but is thought to be associated with many factors, especially local factors. The available evidence suggests that the severity of caries in patients with enamel defects is positively correlated with the degree of enamel defects [4]. Epidemiologic results revealed a high prevalence of rampant caries in the oral cavity of patients with Sjogren’s syndrome (SS) [5]. However, common bad dietary habits, such as drinking many fizzy drinks and eating too many sweet foods, have been found for many rampant caries patients. Studies have shown that taxing on sugar-sweetened beverages and nonessential energy-dense food to discourage the consumption of these products, effectively reduce the incidence of dental caries in local people [6]. Currently, the etiology of rampant caries requires further study.

Microbes are a key factor in caries. For a long time, knowledge on the oral microbial community has been limited due to complicated culture and controversial results. As more than half of the oral bacteria are non-culturable bacteria, it is difficult to recognize the full microbial profile of the mouth [7]. In recent years, the combination of new sequencing technology and information analysis technology has enabled the study of oral microbial communities and the rapid development from low-resolution single-species surveys to high-dimensional community studies [8]. Numerous studies have revealed the impact of oral microbiome on oral and systemic health, and explored its role in disease diagnosis and treatment. For example, some personalized treatments taking into account the systemic risk of the patients has provided new therapeutic strategies in periodontal and caries pathologies preventions, using new professional protocol or domiciliary use of probiotics [9, 10].

With the implementation of the Human Microbiome Project, the characteristics of the oral microbiome have been gradually revealed. The oral cavity is home to a wide variety of microbes, including more than 700 prevalent taxa [11], which contribute significantly to oral health [12].The oral microbiome is unique at different sites between and within individuals due to physical and biological factors. The composition of functionally active microorganisms showed large inter-individual variation according to different states (healthy/diseased) [13]. The oral microbiome colonized on different media surfaces (middle) showed significant space specificity. For example, the microbes that compose dental plaque, mucosa, and saliva are significantly different [14]. Huse et al. [15] compared and analyzed the microbiome at 8 oral sites, including saliva, supragingival plaque, hard palate, palatine tonsil, tongue dorsum, buccal mucosa, subgingival plaque, and keratinized gingiva, for 200 healthy individuals. They found that *Corynebacterium* is diversely distributed in different areas of the mouth, which indicates that different habitats of oral microorganisms have different diversity characteristics.

Oral microbiota dysbiosis can cause oral diseases [16]. Even after symptom resolution, the relative abundance of some bacterial species differed from that of healthy individuals [17]. The composition of the oral microbiota is influenced by a variety of factors, including host diet, inflammatory response, systemic disorders, and habits such as alcohol consumption, smoking, and alcohol consumption. This dynamic ecosystem may cause dysbiosis of oral microorganisms, thus providing opportunities for the occurrence and development of dental and periodontal diseases. Periodontal disease is caused by dysbiosis of the subgingival microbial community. These bacteria drive the process of periodontal disease by orchestrating restructure of the microbiota and promoting inflammation. Dental caries is caused by a high dietary intake of carbohydrates, which leads to more acid production by microorganisms, a decrease in salivary pH, and encourage outgrowth of aciduric and acidogenic species, including Streptococcus mutans and Lactobacillus [18]**.** In this scenario the long-term therapeutic domiciliary use of chlorhexidine can make it worse. The local microbiota naturally tends to return to its initial composition three hours after the antibacterial action of 2% CHX wears off. Even worse, the balance could shift toward more acidic conditions [19, 20]. While the use of ozone therapy or coadjuvant gel could help not just the healing of the mucosal trauma but also to modify the oral microbiota shifting in an healthy one [21, 22].

Based on the existence of numerous variable sequences, 16S rRNA genes can accurately identify bacteria. Sequencing a limited number of clones from each sample provides quantitative information on the relative abundance of each organism in a community [23]. To date, substantial studies have explored the changes in oral microbial diversity and composition in patients with various types of oral diseases based on 16S rRNA genes. Linchen [24] reported that there were no significant differences in biomass, species richness, and species diversity among patients with root caries compared with healthy controls. However, significant differences were found in the beta diversity. *Propionibacterium acidifaciens* and *Streptococcus mutans* were found to be most associated with root caries, which provided a basis for further elucidation of the microbial etiology of root caries in elderly people. In addition, the characteristics of the oral microbiome in patients with and without dental caries were analyzed by [25] using 16S rRNA sequencing. It has even been shown that 16S rRNA gene sequencing analysis of saliva samples collected before caries onset can be used to predict the caries risk of caries-free children [26]. Sequencing, based on 16 s rRNA genes, has been extensively validated and shown to be accurate and useful for oral microbiome studies [27]. Further, oral specimens are easy to handle, sample, and analyze owing to their ability to be non-invasively collected. In addition, oral bacterial markers are relatively stable in individuals, and the diversity of oral microbial communities in the same individual at different time points is significantly lower than that in the intestinal tract, skin, and other body parts [28]. Therefore, oral microflora is a valuable biomarker for disease surveillance and stratification. Scholars have analyzed the oral microbial flora of patients with SS, and reported cases of rampant caries microorganisms [29]. However, some previous studies have not analyzed statistically significant samples [29].

Saliva is responsible for sugar clearance from the mouth, and acid clearance from dental plaque, which will slow down the occurrence and development of dental caries. And Saliva's supersaturation with respect to tooth mineral allows remineralization of teeth with early carious lesions [30]. Microorganisms, electrolytes, proteins, and peptides in saliva can all be used as biomarkers of caries [31]. Caries is driven by the dysbiosis of dental biofilms adhering to tooth surfaces.Under overexposure to dietary carbohydrates, dental plaque forms an acidic microenvironment that demineralizes enamel, leading to permanent tooth damage [32]. It has been reported that the microbial composition of dental plaque varies in people with different caries status [33–35]. The analysis of dental plaque microorganisms is expected to be used for clinical caries status monitoring in the future.

Dental plaque adheres to the tooth surface, and the protection of the matrix makes the composition of the internal flora more stable [36]. But clinically, saliva would be more readily available [37]. This study performed a case–control analysis of saliva and plaque microbiota obtained from the oral cavity 1) to compare the structure and composition characteristics of the oral microbial community in rampant caries patients with those of normal people and common caries patients; 2) to determine whether the distribution characteristics of oral microorganisms in patients with different clinical features of rampant caries (including SS and poor dietary habits) vary; 3) to determine whether saliva can be used as a reliable and stable oral secretion for the screening of rampant caries by comparing the distribution of bacteria in saliva and plaque; 4) to screen out the bacteria with significant differences between patients with rampant caries and normal individuals at the phylum and genus levels; and 5) to screen out the metabolic pathway that may play an important role in the process of rampant caries through functional prediction, to further clarify the cause of rampant caries, and to identify the target in the relevant metabolic pathway. These findings can provide a basis for clinical prevention and treatment.

## Methods

### Ethic statement

All participants provided written informed consent for this institutionally approved study. Approval for this study was obtained from the Nanjing University Affiliated Stomatological Hospital Committee. The study was registered in a clinical registry under the registration number, “chiCTR1800017220”.

### Sample size

This study considered (95% CI) and 90% power to estimate the sample size. The abundance of Veillonella was used as the index [38]. For this purpose, mean ± SD (abundance of Veillonella) value of control group (HP) 0 ± 0 and mean ± SD value of Sjogren’s Syndrome group (SS) 0.00353 ± 0.00228 respectively were taken. Therefore, mean of control group (µ1) = 0, mean of case group (µ2) = 0.00353 and average standard deviation of control and intervention group (ϭ) = 0.00228. Using following formula, Sample Size (n) = (2 ϭ2 (zα/2 + zβ/2)2)/(µ1 − µ2)2, the sample size was calculated as 9. Considering 20% attrition rate total sample size was increased to 12 in each group. The sample size was expanded to 15 participants per group to account for the requirement that samples pass a quality test before 16S sequencing.

### Participants

The participants were from Nanjing University Affiliated Stomatological Hospital. Individuals (self-aware, no mental illness, no screening for restricted mobility, and behavioral disorders) were between 16 and 70 years old.

In this study, the sample size was calculated based on the preliminary experiment and the references. The sample size was estimated according to the calculation method for clinical studies, and based on a review of studies on 16s rRNA sequencing in oral samples or patients with caries. Finally, 15 samples were collected from each group, and a total of 60 subjects were sequenced [34, 39–41]. Thirty patients with rampant caries were included. These individuals had at least five or more caries on their teeth. Fifteen of the rampant caries cases were associated with Sjogren’s syndrome (RC-SS) while 15 were associated with the intake of a high-carbohydrate diet (RC-HD). Another 15 patients with one or two dental caries (DC group) and 15 healthy persons without dental caries (HP group, healthy person) were included in this study. None of the participants had diagnosable periodontitis, gingivitis, and pulpitis; no obvious lesions in the oral mucosa; no oral diseases, such as salivary glands; no severe systemic disease; and no history of genetic disease.

An international multidisciplinary team of experts developed the consensus criteria for primary SS using guidelines from the American College of Rheumatology (ACR) and the European League Against Rheumatism (EULAR). The ACR/EULAR 2016 classification criteria for primary SS could be used to evaluate patients who met the inclusion and exclusion criteria. Patients who scored 4 or more points could be diagnosed with SS, and can be included in the group of rampant caries-SS. Patients who are suspected of SS were required to visit the immunology department of the top three hospitals for diagnosis before enrollment. Patients who could not be diagnosed with SS can be included in the non-SS group [42, 43].

Rampant caries patients with special dietary habits are those who consume excess sugar every day or consume carbonated beverages for long periods. Patients on a high-sugar diet must meet at least two of the following criteria: 1) Sweet tooth (such as consumption of biscuits, chocolate, cakes, candy, ice cream, carbonated drinks, milk, etc.; 2) Prefer to eat before sleeping (and do not brush teeth after eating), at least 3 times per week; 3) Eat dessert at least three times per day (except dinner); and 4) Drink carbonated drinks or sweet/high-sugar drinks at least three times per week.

For the ordinary caries group, the patients were required to have no more than two caries teeth in the oral examination. At least one of the caries should have been present for more than one year. Further, other teeth should not have received dental treatment, such as cavity filling repair and root canal treatment.

The 60 participants were characterized into four groups:

(1) Rampant caries (DMFT ≥ 5) patients with Sjogren’s syndrome (RC-SS, *n* = 15).

(2) Rampant caries (DMFT ≥ 5) patients with a high-sugar diet (RC-HD, *n* = 15).

(3) Dental caries patients (1 < DMFT < 2) (DC, *n* = 15).

(4) Healthy persons with no caries (DMFT = 0; HP, *n* = 15).

### Sample collection

All tissue samples were collected by an endodontic specialist to ensure sample consistency.

#### Saliva sample collection

Unstimulated saliva samples (2 mL) were collected in a saliva collection tube (MoBio tubes). Eating, drinking, especially acid irritants, and mouthwash were not allowed 2 h before sampling. The cheeks were gently rubbed for 30 s to enable saliva accumulation in the patient’s mouth before sampling [42, 43]. Finally, all samples were transported on ice to the laboratory for DNA extraction.

#### Supragingival plaque collection

Supragingival plaque samples were collected from each participant according to the Manual of Procedures for NIH Human Microbiome Project (http://hmpdacc.org/resources/tools_protocols.php), with minor modifications. All individuals were required to avoid brushing their teeth or using bactericidal oral cleaning products within the 24 h before sampling. Eating or drinking was also prohibited for 2 h before collection. Before the start of the collection, the participants were instructed to rinse their mouth with water. The sampling site was isolated with cotton rolls and blown dry with gentle airflow to avoid saliva contamination. Thereafter, a sterile Gracey curette was used to collect accumulated supragingival plaque from the buccal surface of the maxillary first molar, or if necessary, from its adjacent teeth or contralateral teeth to avoid gingival bleeding. The supragingival plaque was collected, immediately transferred to a sterile collection tube with sterile buffer (MoBio buffer), and placed on ice. Finally, all samples were transported on ice to the laboratory for DNA extraction [44, 45].

### Miseq 16S Amplicon sequencing based on the Illumina platform

#### Genomics DNA extraction

Total genomic DNA was extracted using the DNA Extraction Kit (DNeasy PowerSoil Kit (100) QIAGEN 12888–100) following the manufacturer’s instructions. Extracted total genomic DNA was diluted to a concentration of 1 ng/μL and stored at -20 °C until further processing. The quality and quantity of extracted DNA were verified using a NanoDrop 2000 spectrophotometer (Thermo Fisher Scientific, Waltham, MA, USA) and agarose gel electrophoresis, respectively. The requirements for the DNA were as follows: (I) electrophoresis detection involves a single band with no obvious or slight towing, no RNA and protein contamination, (II) DNA concentration ≥ 10 ng/μL, total DNA ≥ 100 ng; (III) OD260/280 = 1.8–2.0. Qualified DNA was used as the template for PCR amplification.

### 16S rRNA gene amplicon libraries and sequencing

Variable regions V3-V4 of the bacterial 16S rRNA gene were amplified with degenerate PCR primers, 515F (5’-GTGCCAGCMGCCGCGGTAA-3’) and 806R (5’-GGACTACHVGGGTWTCTAAT-3’) [46]. PCR amplification was performed in a 50-μL reaction containing 30-ng template, fusion PCR primer, and PCR master mix. The PCR cycling conditions were as follows: 95 °C for 3 min, 30 cycles of 95 °C for 45 s, 56 °C for 45 s, 72 °C for 45 s, and a final extension for 10 min at 72 °C. The PCR products were purified using Agencourt AMPure XP beads and eluted in elution buffer. Libraries were qualified using the Agilent Technologies 2100 Bioanalyzer. The validated libraries were sequenced on the Illumina MiSeq 2500 platform (BGI, Shenzhen, China) following the standard Illumina pipelines, and 2 × 250 bp paired-end reads were generated [47].

### Sequence analysis

Cluster preparation and sequencing were performed using the qualified libraries. Raw sequences were assigned to samples based on their barcodes using QIIME 2.6 [48]. The libraries were denoised and grouped by sequence variants using dada2 1.2.2 [49]. After optimization, the tags were clustered into operational taxonomic units (OTUs), the abundance of which indicates the species richness of the sample, using USEARCH (v7.0.1090) software with 97% similarity [50]. Species annotation and classification of OTUs were carried out by comparing OTUs with the Human Oral Microbiome Database (HOMD) [51]. The abundances of OTUs in the samples were then standard normalized, and the microbial taxa (phylum, class, order, family, and genus) were classified using the Rhea pipeline before statistical comparison [52].

A Venn diagram was used to reveal the overlap of OTU between samples. Principal Components Analysis (PCoA) was performed using R (v3.1.1) software [53]. The Alpha diversity and Beta diversity distances were calculated. The calculation formula for each index was derived from http://www.mothur.org/wiki/Calculators.

To obtain the species classification information corresponding to each OTU, the RDP classifier was used for the taxonomic analysis of the OTU representative sequences, and the community composition of each sample was counted at the genus level of the genus family. The Wilcoxon rank-sum test (for two groups) and the Kruskal–Wallis test (for more than two groups) were used to assess the significant differences between the two groups of samples and the FDR value for the p-value was calculated using software R (v3.4.1). The unweighted pair group method with arithmetic mean (UPGMA) was employed as the clustering method, and linear discriminant analysis (LDA) analysis was performed using LEfSe software (https://huttenhower.sph.harvard.edu/galaxy/).

This study used rampant caries enrichment, common caries enrichment, and control-enriched OTUs to generate abundance-based correlation networks to detect synergies between groups under different health conditions. Pearson’s correlation coefficient between the OTUs after logarithmic ratio transformation was calculated. This study used the phylogenetic investigation of communities by reconstructing the unobserved states (PICRUSt, http://huttenhower.sph.harvard.edu/galaxy) to predict community function using the Kyoto Encyclopedia of Genes and Genomes (KEGG) database [54, 55].

Statistical analyses and plots were performed using the QIIME (v1.80) software. The correlation network was visualized using Cytoscape and R v3.4.1. Statistical significance was set at p < 0.05.

### Statistical analysis

Clinical and demographic data were compared using analysis of variance (ANOVA) or the nonparametric Kruskal–Wallis rank-sum test, depending on the normality of the data, using GraphPad Prism 8. Differences were considered significant at p values of < 0.05.

## Results

### Clinical features of patients with rampant caries

**Fig. 1:**
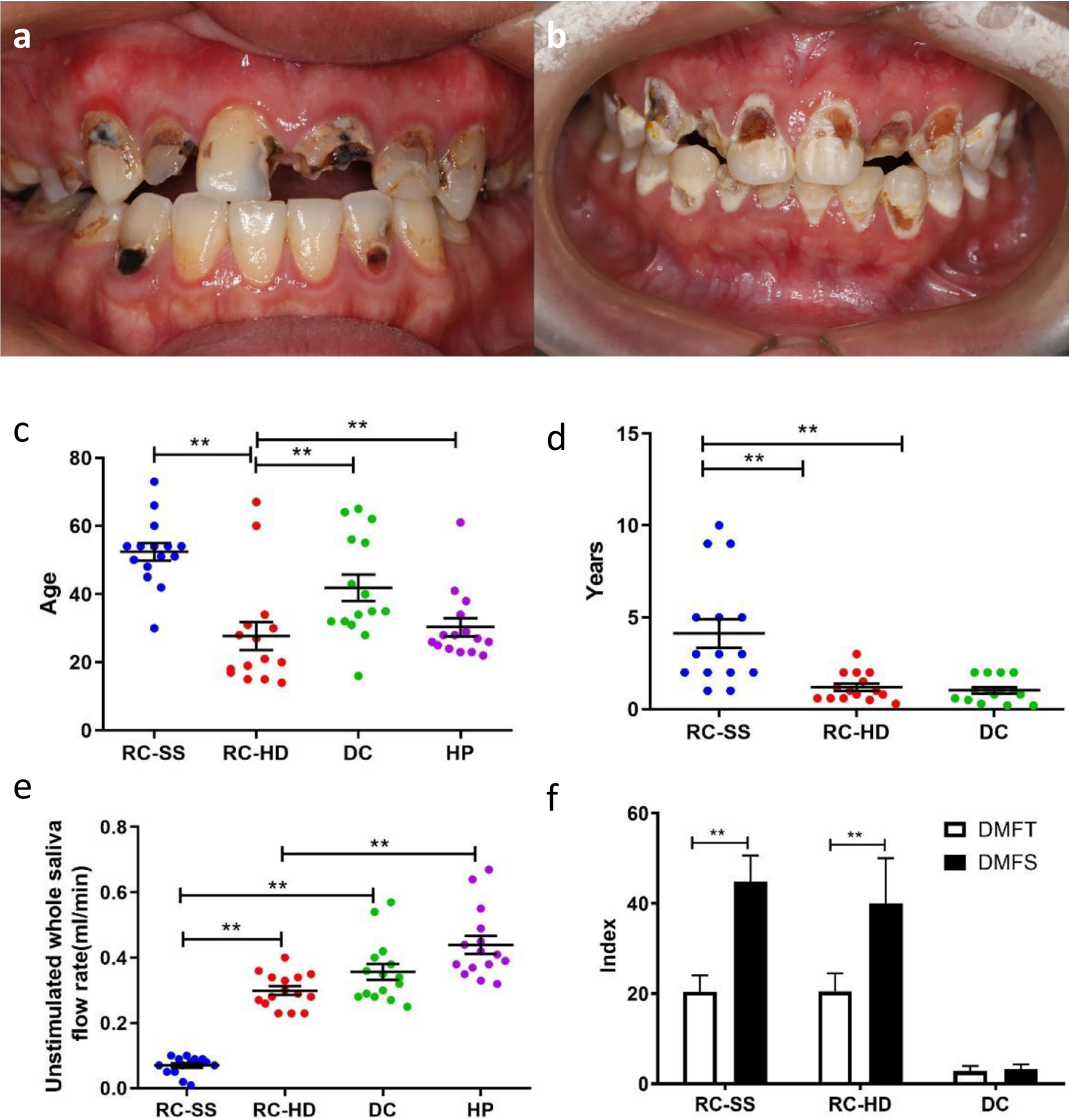
The clinical feathers of the rampant caries patients and the controls. **a**. A patient with Sjogren's Syndrome; **b** A patient with high-sugar diet. **c**. The ages of the four groups. There are significant differences between RC-SS and RC-HD group. **d**. the time from the caries began to be detected. There are significant differences between RC-SS and RC-HD group, and also between RC-SS and DC group. **e**. The saliva flow rate of the RC-SS group is lower than the other three groups. **f**. The results of DMFS and DMFT. (* *P* < 0.05, ** *P* < 0.01)

The age, age of onset, decayed missing filled teeth (DMFT) and decayed missing filled surface (DMFS) of the four groups were compared (Fig. 1). The patients with SS (Fig. 1a) were found to have hard caries, while the patients with high sugar diet (Fig. 1b) had soft caries. A significant difference (*P* < 0.01) in age was found between the RC-SS group (48.90 ± 2.51) and RC-HD group (20.88 ± 1.80) (Fig. 1c). The timing of the initial clinical detection of dental caries was significantly different (*P* < 0.01) between the RC-SS group (6.60 ± 0.98) and the RC-HD group (1.61 ± 0.28) or DC group (0.96 ± 0.17) (Fig. 1d). The saliva flow rate of the RC-SS group is lower than the other three groups (Fig. 1e). The DMFT results revealed that RC-SS group (20.40 ± 1.14) and RC-HD group (20.50 ± 1.41) had significantly higher DMFT than the DC group (2.79 ± 0.318); however, there was no significant difference between the two groups. Based on the DMFS results, the RC-SS group (44.80 ± 1.86) and RC-HD group (40.00 ± 3.55) had significantly higher DMFS than the DC group (3.21 ± 0.28), and DMFS was significantly higher in patients with rampant caries than in patients with DMFT. Such finding indicates that rampant caries have characteristics of multiple concurrent caries (Fig. 1f).

### Phylogenetic alterations of the microbial community

**Fig. 2:**
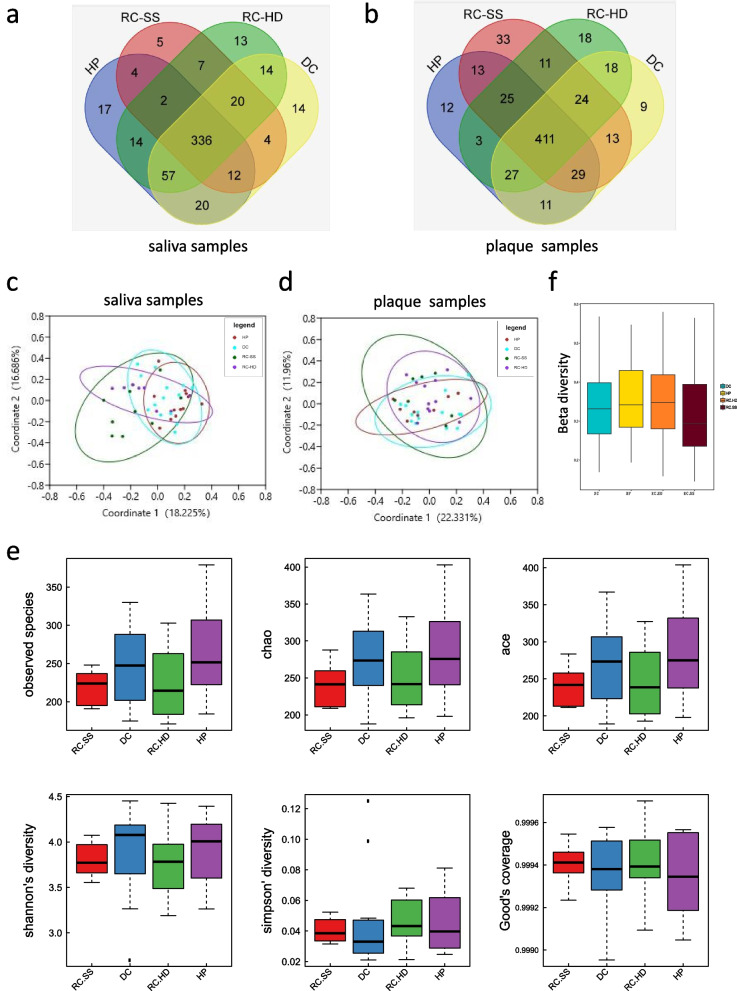
Principal components analysis and diversity analysis. Number of common and unique OTUS by Venn (Fig. 2a, b) and OUT Principal coordinate Analysis (PCoA) with Bray–Curtis algorithm (Fig. 2c, d). The Venn diagram showing shared and unique OTUs at 95% identity among the four groups of saliva (Fig. 2a) and plaque samples (Fig. 2b). Observed species, chao, ace, shannon’s diversity, simpson’s diversity and good’s coverage were used to as index of alpha diversity (Fig. 2e). The Beta diversity index (Fig. 2f) between groups was tested by the multi-sample comparison Kruskal–Wallis Test (Kruskal. Test in R). There were no significant differences among four groups (*P* > 0.05)

A total of 44 saliva samples (10 RC-SS, 8 RC-HD, 14 DC, 12 HP) and 41 plaque samples (7RC-SS, 12RC-HD, 14 DC, 8 HP) passed quality inspection and were subjected to 16S rRNA amplicon library sequencing. After data processing, the saliva samples yielded 1,523,220 high quality sequences, with an average of 34,618 per sample. A total of 1,889,360 high quality sequences were obtained from plaque samples, with an average of 46,082 sequences per sample. A total of 658 OTUs of saliva samples and 534 OTUs of plaque samples were obtained by OTU clustering and filtration of clean tags. The out results are shown in Supplementary Table 1. The Venn diagram highlights the shared and unique OTUs at 95% identity among the four groups of saliva (Fig. 2a) and plaque samples (Fig. 2b).

### Diversity analysis and bacterial community structure

Figure 2c and d show the results of PCoA using the Bray–Curtis ordination based on the OTUs classification across the saliva and plaque samples. Based on the saliva samples, the RC-SS group exhibited clear segregation in community structures among the groups, occupying the first 2 principal components. However, based on the plaque samples, the RC-SS and RC-HD microbiota overlapped with some HP and DC microbiota. Observed species, Chao, ace, Shannon’s diversity, Simpson’s diversity and Good’s coverage were used to derive the index of alpha diversity (Fig. 2e). The Beta diversity index (Fig. 2f) between groups was determined by multi-sample comparison Kruskal–Wallis Test (Kruskal. Test in R). There were no significant differences among the four groups (*P* > 0.05).

### Bacterial abundance and distribution

**Fig. 3:**
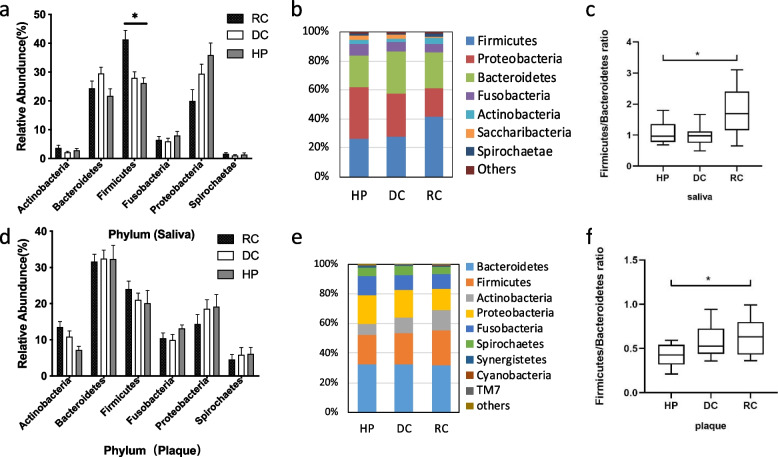
The relative abundance of phylum for saliva (Fig. 3a, b, c) and dental plaque (Fig. 3d, e, f) samples in RC, DC and HP groups. In saliva, the *Firmicutes* of the rampant caries group significantly increased. In plaque and saliva, the F/B ratio of rampant caries group was significantly higher than that of the normal population, and the mean value of saliva was higher than that of plaque. (* *P* < 0.05)

**Fig. 4:**
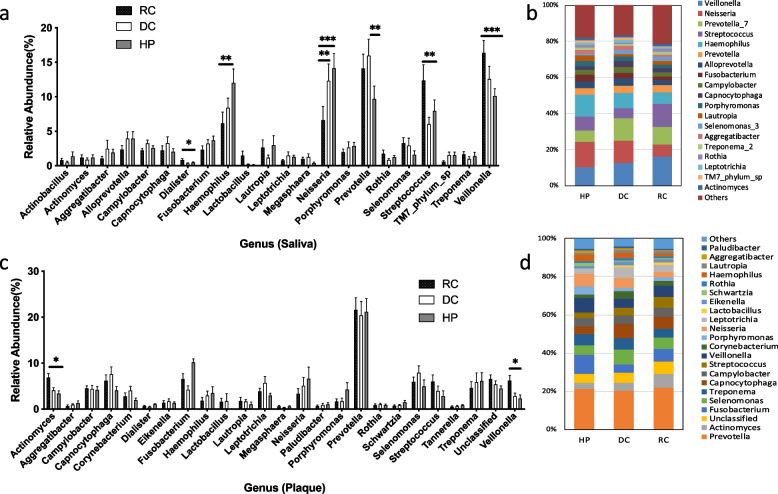
The relative abundance of genus for saliva (Fig. 4-a,b) and dental plaque (Fig. 4-c,d) samples in RC, DC and HP groups. In the level of Genus, top 10 are *Prevotella*, *Veillonella*, *Streptococcus*, *Actinomyces*, *Fusobacterium*, *Capnocytophaga*, *Selenomonas*, *Leptotrichia*, *Campylobacter*. In saliva samples, *Veillonella* and *Streptococcus* of group RC is higher than DC and HP. *Neisseria* and *Haemophilus* are significant reduced. The overall expression of *Prevotella* expressed high both in saliva and plaque. It is increased significantly for RC group in saliva, but there was no significant difference in plaque among the three groups. (* *P* < 0.05, ** *P* < 0.01, *** *P* < 0.01)

Microbiota in saliva and plaque samples were identified at all taxonomic levels, and bacterial composition changes in rampant caries, dental caries, and healthy controls were identified. The relative abundances at the phylum and genus levels are shown in Fig. 3 and Fig. 4, respectively. In saliva samples (Fig. 3a, b), *Firmicutes, Proteobacteria, Bacteroidetes, Fusobacteria, Actinobacteria, Saccharibacteria,* and *Spirochaetae* constituted the majority of the predominant salivary microbiota at the phylum level. In the plaque samples (Fig. 3d, e), the most abundant phyla were *Bacteroidetes, Firmicutes, Proteobacteria, Actinobacteria, Fusobacteria,* and *Spirochaetes*. In the saliva of patients with rampant caries, the relative abundance of *Firmicutes* increased significantly at the phylum level (*P* < 0.05). In plaque and saliva, the *Firmicutes/Bacteroidetes* ratios (Fig. 3c, f) of the RC group were significantly higher than those of the normal population, and the mean value of saliva was higher than that of plaque (*P* < *0.05*). The proportions of the top 20 species at the genus level are shown in Fig. 4b, d. *Streptocpccus, Veillonella, Prevotella,* and *Dialister* increased*,* while *Neisseria and Haemophilus* were reduced in saliva samples from rampant caries patients (*P* < 0.01) (Fig. 4a). *Veillonella* increased in the plaque samples of the RC group (*P* < 0.05) (Fig. 4c).

### Microbiota compositions and biomarkers for RC-HD and RC-SS

**Fig. 5:**
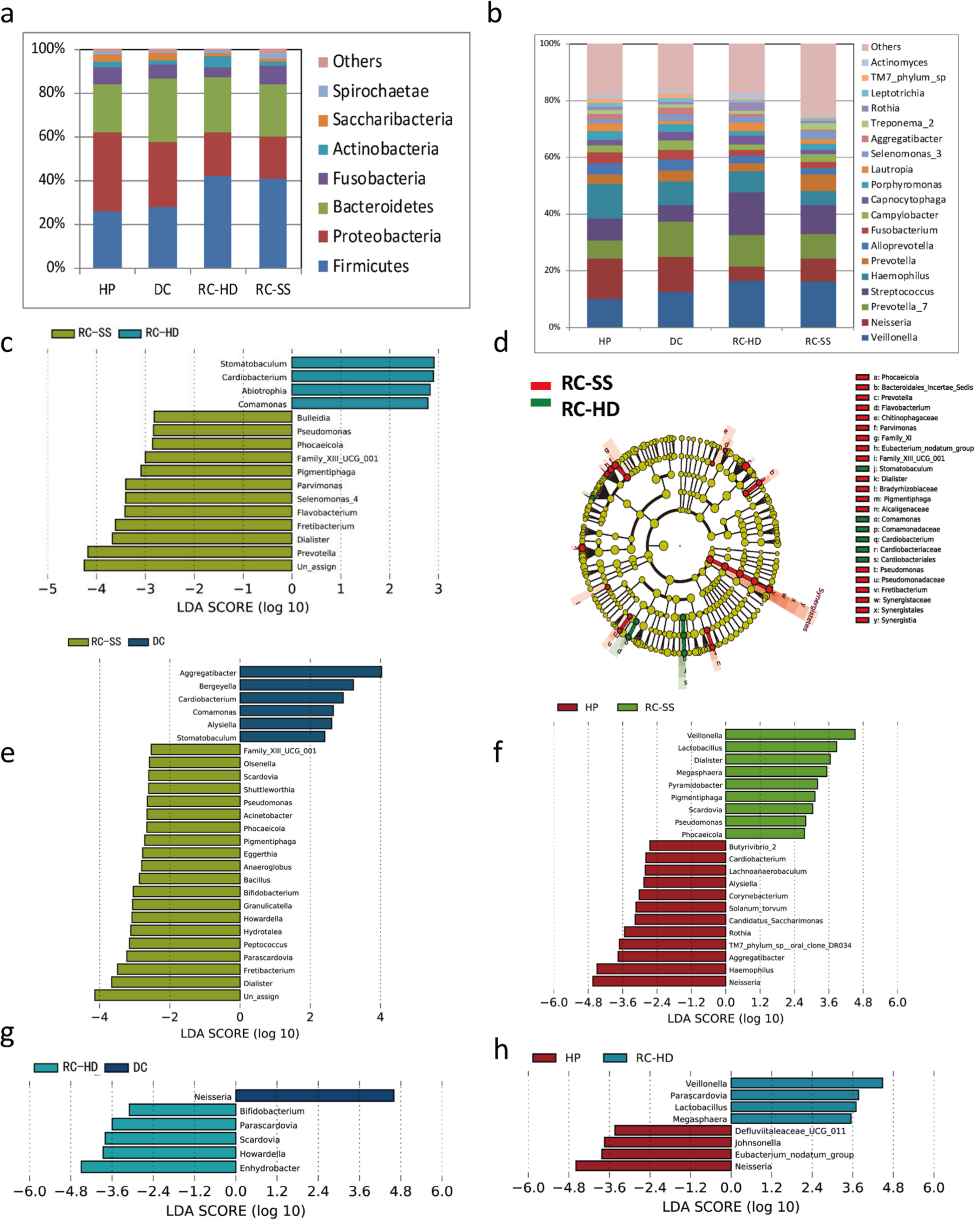
Comparisons of the phylogenetic structure and composition among the saliva microbial communities from four groups. Species abundance in the level of phylum (Fig. 5a) and genus (Fig. 5b) in RC-HD, RC-SS, DC and HP groups. Biomarkers of four groups in saliva samples with LEfSe analyzes showing as cluster graph (Fig. 5d) and LDA pictures (Fig. 5c, e, f, g, h)

To compare the oral microbiota community of rampant caries with different etiologies (SS or HD), oral samples from patients with rampant caries of different causes were collected and detected separately (called RC-SS and RC-HD). Figure 5 showed comparisons of the phylogenetic structure and composition among the saliva microbial communities from four groups. According to the top abundance species at the level of phylum (Fig. 5a) and genus (Fig. 5b), the core microorganisms included *Veillonella, Neisseria, Prevotella, Streptococcus, Haemophilus, Fusobacterium, Campylobacter* and *Capnocytophaga.* Biomarkers with LEfSe analyzes showing as cluster graph (Fig. 5d) and LDA pictures (Fig. 5c, e, f, g, h). For saliva samples, *Prevotella, Dialister* and *Fretibacterium* significant enriched in the RC-SS group.

**Fig. 6:**
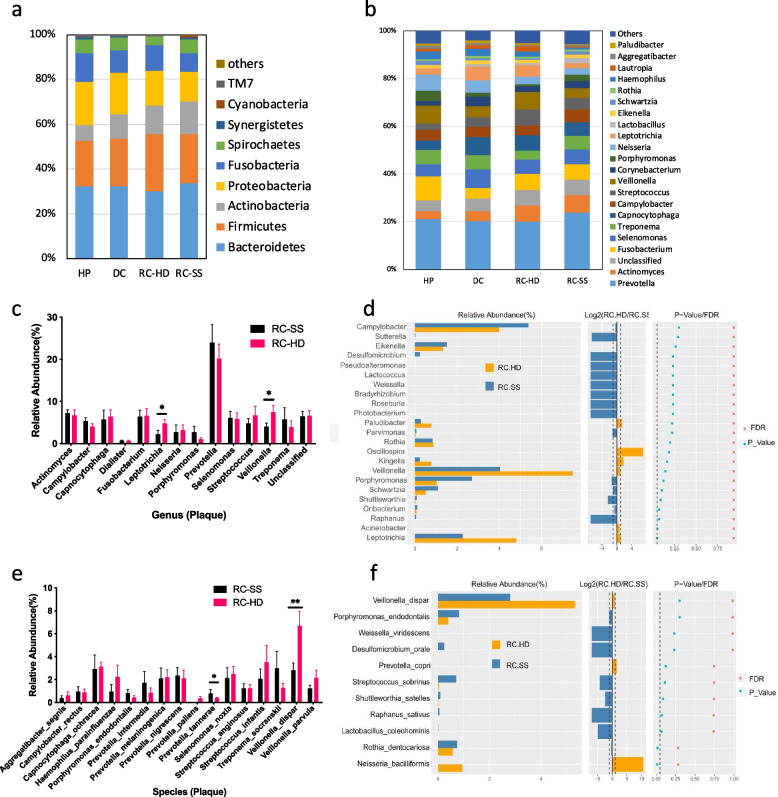
Comparisons of the phylogenetic structure and composition among the plaque microbial communities from four groups. Relative abundance in the levels of phylum (Fig. 6a), genus (Fig. 6b) among the four groups. Relative abundance screening of major species for plaque microbial communities with Wilcoxon rank-sum test between RC-HD and RC-SS groups. Top floras in the level of genus (Fig. 6c, d) and species (Fig. 6e, f). At the level of species, *Veillonella dispar* is higher in group RC-HD (* P < 0.05, ** *P* < 0.01)

According to the results of plaque samples, the core microorganisms included *Prevotella, Actinomyces, Fusobacterium, Selenomonas, Treponema, Capnocytophaga, Campylobacter, Streptococcus,* and *Veillonella* (Fig. 6a, b). Relative abundance screening of major species between RC-HD and RC-SS patient plaque microbial communities using the Wilcoxon rank-sum test is depicted in Fig. 6c, d, e, f. The level of *Veillonella* and *Leptotrichia* in the RC-HD group was significantly higher than that in the RC-SS group (*P* < 0.05) (Fig. 6c). *Prevotella tannerae* were higher in the RC-SS group than the RC-HD group. However, in the plaque samples, *Veillonella dispar* was higher in the RC-HD group than the RC-SS group (*P* < 0.05) (Fig. 6e).

**Fig. 7:**
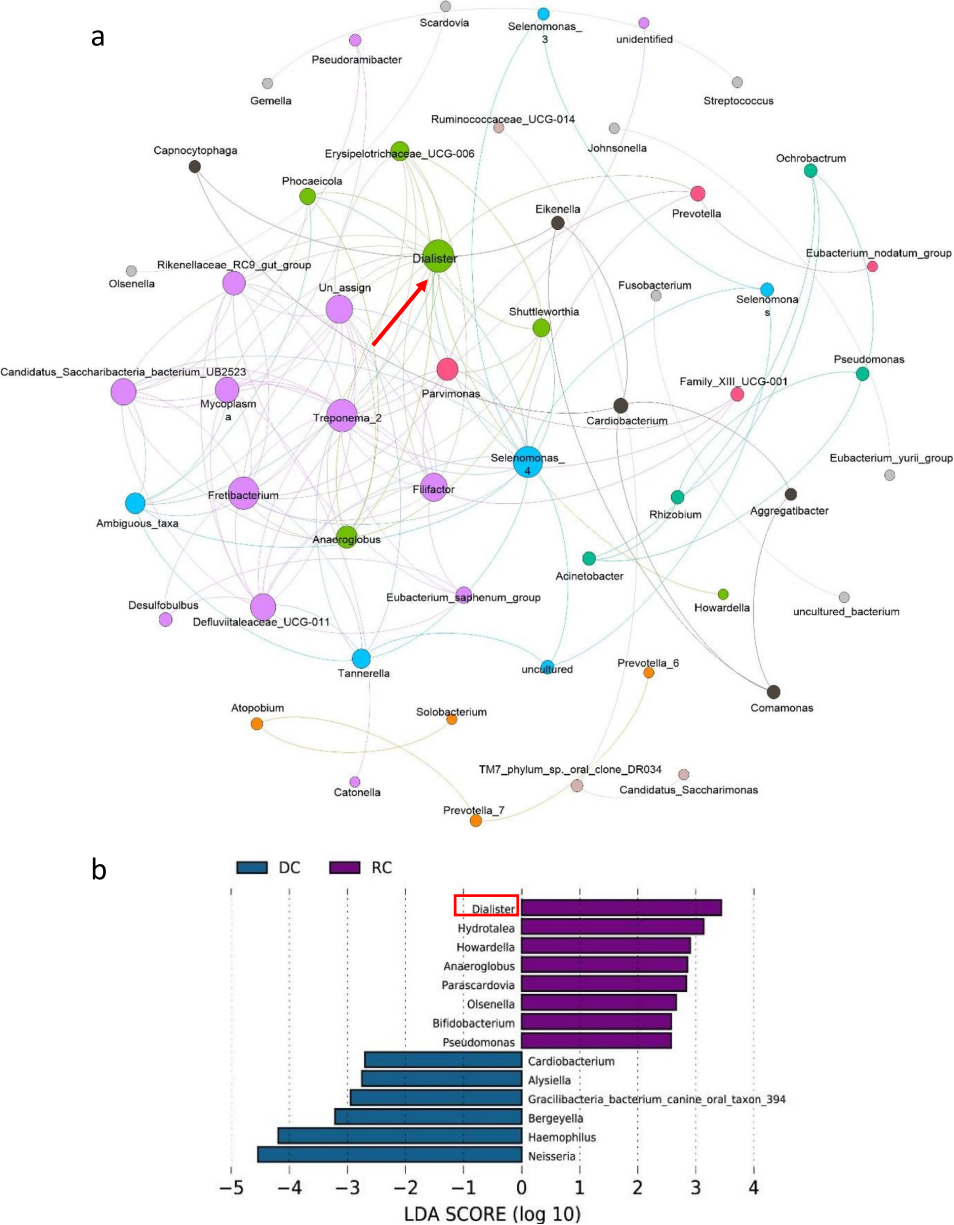
Network analysis of the co-occurence patterns of four groups detected dominant genera (Fig. 7a). The nodes are color-coded to reflect the modularity classes. The size of each node is proportional to the number of connections. Results of Linear Discriminant Analysis and screening based on LDA Score showed that Dialister was significantly enriched in the the rampant Caries patients (Fig. 7b). In the Network analysis Dialister was selected as one of the representative markers of those associated microbial species in salivary

Network analysis of the co-occurrence patterns of four groups detected 232 OTU profiles and 251 functional abundances was showed in Fig. 7 and Fig. S2. As shown in Fig. 7a, dominant genera were detected by network analysis of the co-occurrence patterns of the four groups. In the network analysis, *Dialister* was selected as a representative marker of the associated microbial species in saliva, which is consistent with the results of LEfSe analysis. Linear discriminant analysis and screening based on the LDA score showed that *Dialister* was significantly enriched in rampant caries patients (Fig. 7b). As showed in Fig. S2, it is believed that some OTU abundance can be used as a biomarker for oral microbial function such as OTU1, OTU5 and OTU47.

### Involvement of differentially abundant families in functional variation

**Fig. 8:**
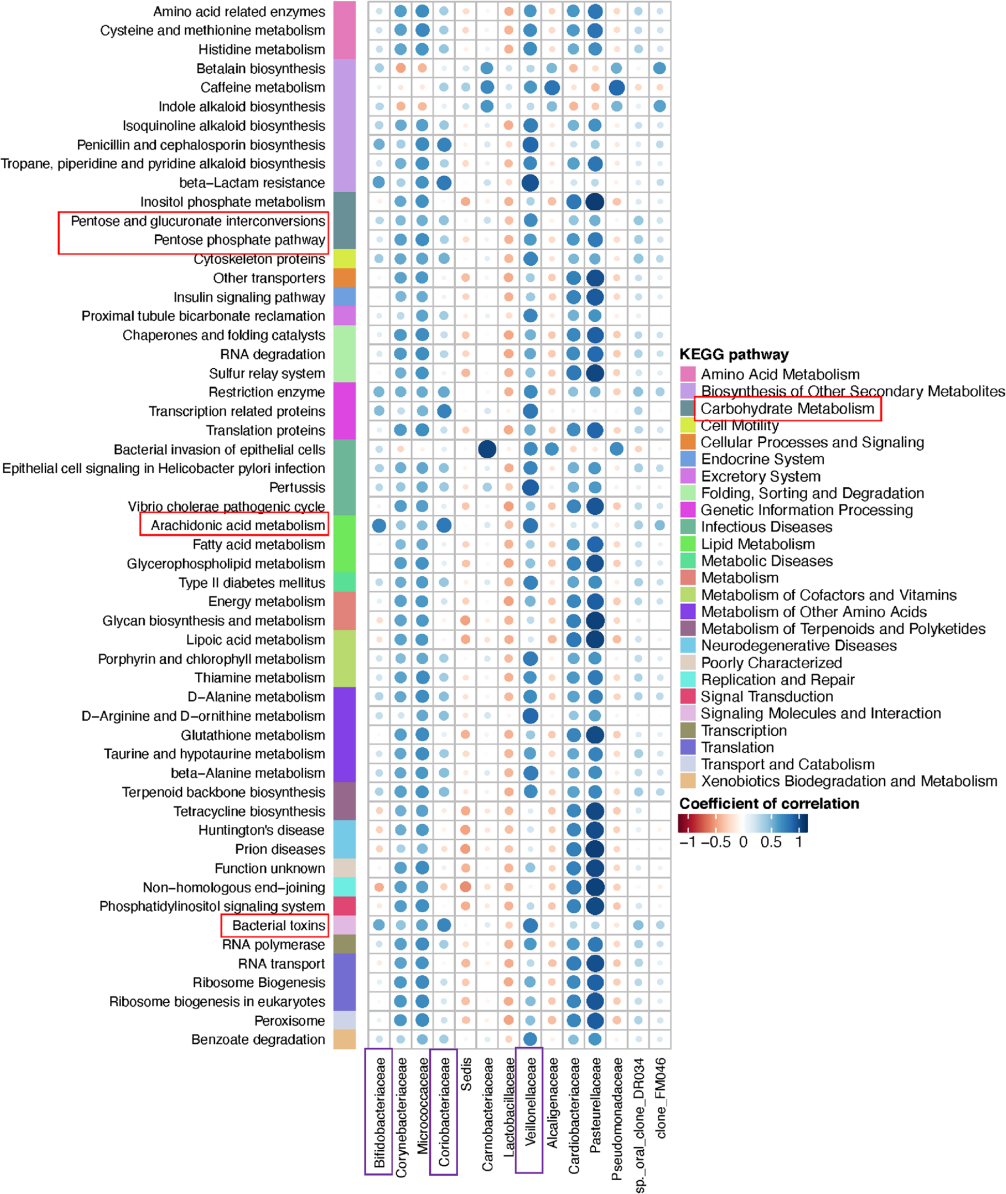
Bacterial function prediction by PICRUSt analysis. Associations between altered families and functions in the rampant caries microbial community and health. The result of KEGG prediction of RC-SS group shows that *Cardiobacteriaceae*, *Pasteurellaceae*, *Corynebacteriaceae* and *Micrococcaceae*, *Veillonellaceae*, *Carnobacteriaceae* exist in the metabolic characteristics significantly. *Veillonellaceae* is related to Carbohydrate Metabolism, such as Pentose and glucuronate interconversions, Pentose phosphate pathway. And *Veillonellaceae*, *Bifidobacteriaceae*, *Coriobacteriaceae* are related with Arachidonic acid metabolism, Bacterial toxins

Another objective of this study was to reveal the functional variation in the salivary microbial community for rampant caries. Therefore, the PICRUSt algorithm with the KEGG database was used to predict the microbiota-derived pathways and compared the functional abundance among the RC-SS (Fig. 8), RC-HD (Fig. S1), and the control group [56]. The functional changes in RC-SS and RC-HD samples included loss of genetic information processes (e.g., replication and repair, transcription, and translation) and basic metabolism (e.g., amino acids, energy, cofactors, and vitamin metabolism) (Fig. 8 and Fig. S1). The results of KEGG analysis of the RC-SS group showed that *Cardiobacteriaceae, Pasteurellaceae, Corynebacteriaceae, Micrococcaceae, Veillonellaceae*, and *Carnobacteriaceae* had significant differences in the metabolic pathways (Fig. 8). *Veillonellaceae* is related to carbohydrate metabolism, such as pentose and glucuronate interconversions and the pentose phosphate pathway, while *Veillonellaceae, Bifidobacteriaceae,* and *Coriobacteriaceae* are related to arachidonic acid metabolism and bacterial toxins.

## Discussion

Systemic autoimmune and inflammatory diseases often manifest as oral lesions in the early stage, such as Sjogren's syndrome with oral mucosal lesions. Early diagnosis by dental clinicians is the key to improve the prognosis [22, 57]. Present study suggests that SS has a negative effect on osseointegration in oral implants [58, 59]. In terms of periodontics, the periodontal parameters, including plaque index (PI) and gingival index (GI), were higher in SS patients than in healthy people, which may be related to the lower salivary flow rate [60]. Dental caries is the most common chronic infectious disease of the oral cavity, and is traditionally thought to be caused by a single pathogen [61]. However, owing to the recent failure of traditional pathogen prevention and control methods to effectively reduce the incidence of caries, people have realized that a single theory cannot completely explain the disease. Accordingly, research on the relationship between microorganisms and the etiology of caries has been gradually transferred from the traditional theory to the microbial ecological balance theory [62]. With the development of detection methods, numerous studies on cavity microbial communities have been carried out in succession. Further, the concept that a group of microorganisms play a key role in the occurrence and development of dental caries has been proposed [41, 63].

Dental caries is a disease induced by dental plaque, which can be described as a community of microorganisms (biofilms). Due to genetic and environmental factors, the oral microbiota undergoes many changes, and homeostasis can be disrupted [18]. As the distribution of bacteria in different parts of the oral cavity is different, the differences in flora from several levels were analyzed. First, patients were grouped according to the degree of caries, and the flora distributions of patients with rampant caries and ordinary caries, and those of normal people were compared. Second, different tissues were sampled to assess the differences in the flora distribution of saliva and dental plaque. Finally, the expression of oral microorganisms for rampant caries with different clinical characteristics was revealed in this study.

The limitations of the samples used in this study are worth discussing. Before sample collection, the sample size for this study was estimated on the basis of similar studies, with an expected sample size of 20 to 30 per group. However, rampant caries is the most serious type of dental caries. The clinical incidence of rampant caries is lower than that of common caries. A total of 60 individuals were included in this study, with 15 individuals assigned to each group. However, only 44 saliva samples and 41 plaque samples passed the quality test and underwent 16S sequencing. Further, many samples did not undergo a quality inspection, which might be related to the long sample extraction time and small sample size. These are the limitations of the study. In the subsequent research plan, clinical samples will continue to be collected and the sample size will be expanded.

In this study, resting or unstimulated total saliva flow rate was measured using the spitting method, which is relatively common and simple, and can be used for the total determination of saliva content. Patients in this study had a small amount of saliva and a 10-min spit test was performed. According to the diagnosis label in SS, a saliva volume < 1 mL/10 min is defined as dry mouth. In summary, the degree of dental caries, chief complaints of dry mouth, and saliva flow test results were the main criteria for study inclusion. These criteria are fundamentally different from those of other studies on SS flora, and serves as one of the highlights of our study.

In this study, patients with a high-sugar diet were selected by the frequency of sugar intake, as the frequency of sugar intake contributes more to caries than the quantity [64]. But it is difficult to determine sugar intake under a set of conditions. In China, carbonated drinks are frequently consumed owing to the extremely high public demand for this type of drink. The average consumption of 500 ml of carbonated beverages daily, equal to more than one bottle (500 ml/bottle), is considered as the basic condition for inclusion in the study. Other participants had a taste for sweets to varying degrees. As the enrolled patients were not managed centrally, they could not unify their diet and had certain limitations. In future studies, the interference of dietary varieties will be further excluded, and the daily sugar intake will be quantified to obtain more accurate results.

In this study, there were similarities and differences in the expression of oral microorganisms in rampant caries with different clinical characteristics. Such finding may be related to the features of disease occurrence. SS, the second most common chronic autoimmune rheumatic disease, mainly involves the exocrine glands, with dry lips as the main manifestation. The constituents and properties of this oral fluid play an essential role in the occurrence and progression of dental caries [31]. Surveys have shown that 47.5% of patients with SS suffer from rampant caries. The cleaning and buffering effect of saliva helps to control the occurrence and development of dental caries. Some scholars [43] found that the SS group had higher microbial diversity than the control group based on high-throughput sequencing analysis. In particular, the number of *Bacteroidetes* and *Actinomycetes* was significantly increased. Kegg-lefse functional difference analysis showed that genes related to immune function were significantly upregulated in the SS group, suggesting an enhanced autoimmune response in SS patients, which is consistent with the view that SS is an autoimmune disease.

In the oral samples of rampant caries patients that consumer a high-sugar diet, *Prevotella, Veillonella, Streptococcus, Actinomyces,* and *Capnocytophaga* were significantly enriched. Another research revealed that the Swedish adolescents consumed sweet snacks, and those with caries activity had *Prevotella, Actinomyces,* and *Capnocytophaga* [41]. The current theory suggests that caries is not caused by a single bacterium or a handful of bacteria, but by a community of microbes [65]. From the perspective of microbial flora theory, the formation of microbial society is related to genetic and environmental factors [66, 67]. The microbial community induces the production of bacterial membrane plaques. Through acid production, sugar decomposition is accelerated, which indicates that sugar, acidic foods, and sticky foods that adhere to the tooth surface for a longer period are risk factors for tooth decay. When sugar or other carbohydrates enter the oral cavity, they are metabolized into acids by microorganisms, which causes the pH of the plaque to decrease, and consequently, the solubility of the hydroxyapatite to increase, thereby inducing tooth demineralization [68]. Based on the WHO guidelines, the intake of free sugars should provide < 10% of the energy intake [69]. For an average adult, 10% of energy from free sugars equates to 50 g or 10 teaspoons of sugar per day [70]. Fortunately, many bacteria, such as *S. mutans*, can metabolize sugars, which can control the occurrence and development of dental decay. The body's natural defense mechanisms, especially saliva washing and neutralization, also play a significant role.

Overall, in this study, there were no significant differences between dental plaque and saliva, which were employed to reflect the bacterial characteristics of rampant caries. Nonetheless, saliva was easy to obtain and the bacterial composition varied slightly between individuals [71]. Saliva is rarely affected by blood, gingival crevicular fluid, and other tissue fluids; therefore, it has more clinical diagnostic significance. According to the results of the study, the 16S test results of saliva and plaque showed that *Veillonella* and *Streptococcus* in the rampant caries group were significantly higher than those in the DC and HP groups, while *Neisseria* was significantly lower. *Rothia* decreases in the plaque and increases in the saliva. The change trends of *Prevotella* were found to be inconsistent. *Prevotella tannerae* decreased significantly in the plaques, *Haemophilus* decreased significantly in saliva but not in plaques, and *Capnocytophaga* increased significantly in plaques, but not in saliva. Some researchers have shown that *Streptococci and Veillonellae* play important roles in the etiology of caries [41].

The mechanism of rampant caries requires further study. During the formation of early dental plaque, *Streptococci* and *Veillonellae* occur in mixed-species colonies. One factor that is assumed to be important in the assembly of these initial communities is co-aggregation (cell–cell recognition by genetically distinct bacteria). Intrageneric coaggregation of S*treptococci* occurs when a lectin-like adhesin on one streptococcal species recognizes a receptor polysaccharide (RPS) on the partner species. *Veillonellae* also co-aggregate with *streptococci* [72]. This study confirms that RPS-mediated intrageneric coaggregation occurs in the earliest stages of plaque formation via bacterial accumulation to form a functional community Altschul [73].

When patients with rampant caries are compared with those with common caries and normal people, the saliva and plaque results were found to be similar, and both the core microorganisms and the different microorganisms were similar. Saliva samples may be considered when sufficient plaque samples cannot be used to detect microbial expression. Indeed, it is better to test saliva and dental plaque samples simultaneously to evaluate and predict the prevalence of dental caries caused by specific factors by microbial distribution.

Dental caries is a dynamic process that depends on the acidification of the environment; therefore, molecular microbiological studies should not only include identification and quantity parameters of microbiome composition, but also metabolic activity. Veillonellaceae is related to carbohydrate metabolism, such as pentose and glucuronate interconversions and the pentose phosphate pathway. These results suggest the involvement of differentially abundant families in functional variation, which provides a theoretical basis for further studies on the metabolic function and mechanism of the interaction of core microbiota in rampant caries. Subsequently, in vitro and animal experiments will be designed for verification.

In this study, Illumina sequencing was used for rampant caries, dental caries, and healthy patients without caries from eastern China. The oral core microbiome and biomarkers of oral saliva and dental plaque were revealed herein. Further, the study (1) classified rampant caries according to the different clinical manifestations; (2) revealed that the results were statistically significant; and (3) comprised adults with rampant caries.

The 16 s rRNA of saliva in patients with rampant caries and KEGG Lefse analysis only enable preliminary detection and identification, thereby serving as a limitation of the study. Future research should further explore the immune mechanism of SS rampant caries and the metabolic mechanism of high-sugar diet rampant caries. The relationship between a high-sugar diet and SS and rampant caries needs to be fully clarified and further discussed. Owing to the difficulty in case collection, the number of patients included in this study was not sufficient to carry out an in-depth investigation of the influence of dietary habits, social status, and other factors. Thus, these limitations need to be addressed in future studies.

### Supplementary Information

**Supplementary Material 1.**

**Supplementary Material 2.**

## Data Availability

All raw sequences were deposited in the NCBI Sequence Read Archive under accession number PRJNA643872. The SRA records will be accessible with the following link after the indicated release date: https://www.ncbi.nlm.nih.gov/sra/PRJNA643872.
