# Supplementary material for: Effects of Sjogren’s syndrome and high sugar diet on oral microbiome in patients with rampant caries: a clinical study
Source: BMC Oral Health. 2024 Mar 21;24:361. doi: 10.1186/s12903-024-04150-8 (PMC10956276; doi:10.1186/s12903-024-04150-8)
Supplement: Supplementary file 2 — Supplementary Material 2. [file 12903_2024_4150_MOESM2_ESM.pdf]

Supplement Figure 1

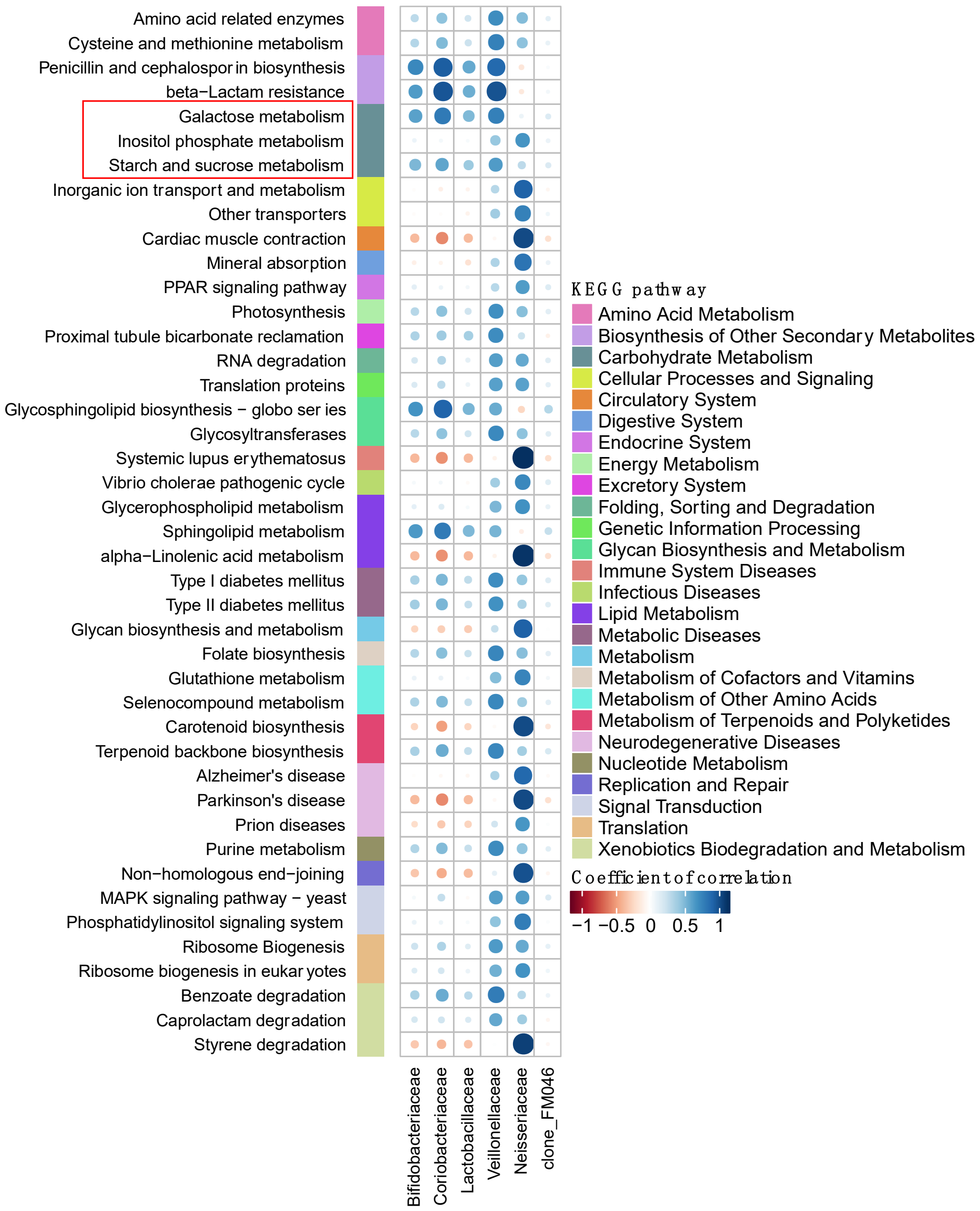

Supplement Figure 1. Bacterial function prediction by PICRUSt analysis. The result of KEGG prediction of RC-HD group shows that *Bifidobacteriaceae, Coriobacteriaceae, lactobacillus, Veillonellaceae* are focus on the pathways of Carbohydrate Metabolism, the Metabolism of Galactose Starch and sucrose increased significantly, but that of *Neisseriaceae* was not obviously.

Supplement Figure 2

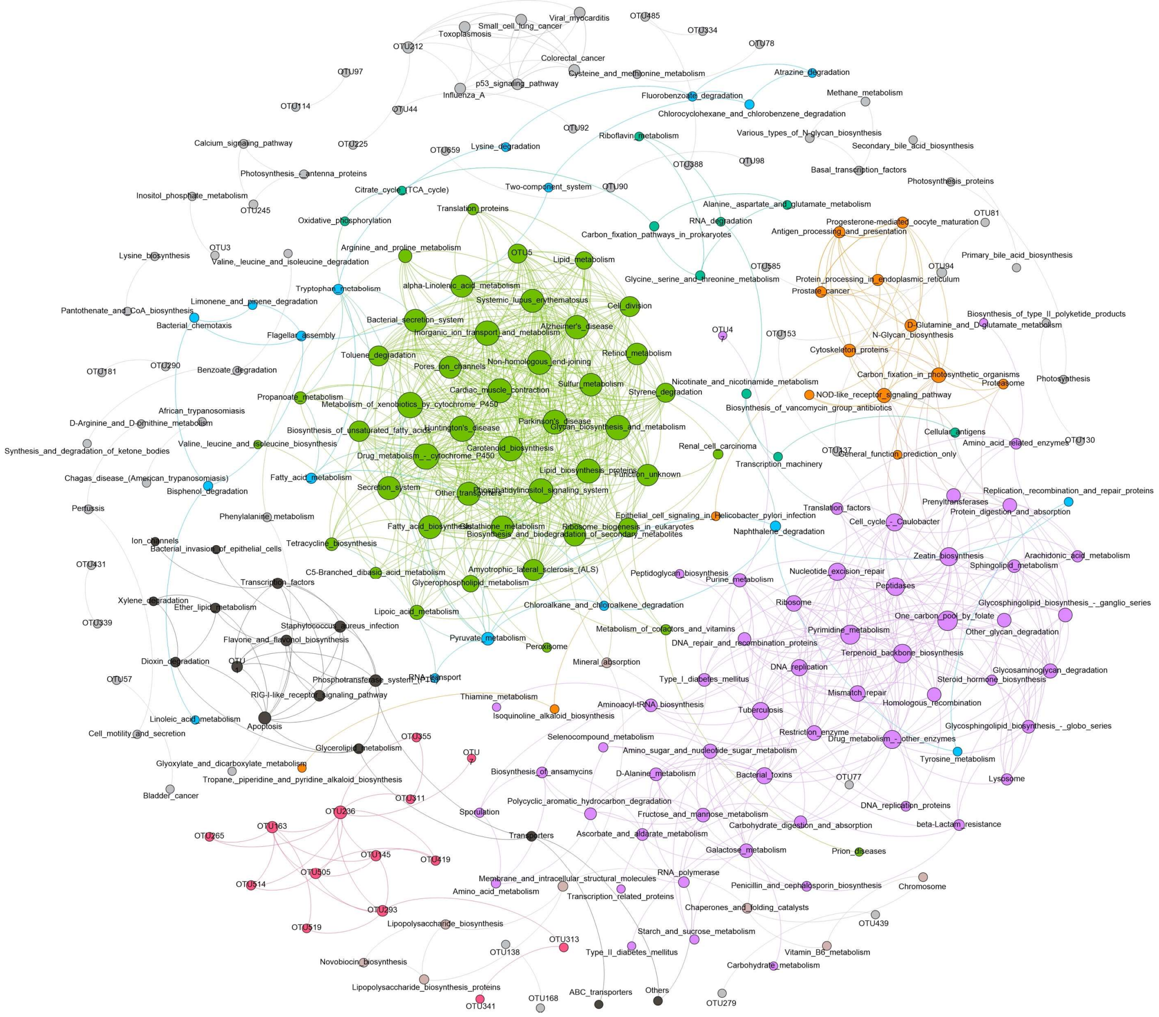

Supplement Figure 2. Network analysis of the co-occurrence patterns of four groups detected 232 OTU profiles and 251 functional abundances. The nodes are color-coded to reflect the modularity classes. The size of each node is proportional to the number of connections. Relevant OTUs and functions are identified in the same color. It is believed that some OTU abundance can be used as a biomarker for oral microbial function such as OTU5, OTU1 and OTU47.
